# Efficacy and safety of COVID-19 inactivated vaccine: A meta-analysis

**DOI:** 10.3389/fmed.2022.1015184

**Published:** 2022-11-07

**Authors:** Xiaoming Li, Xia Yang, Zong Ning

**Affiliations:** ^1^Department of General Practice, School of Graduate Studies, Guangxi Medical University, Nanning, China; ^2^Department of General Practice, The First Affiliated Hospital of Guangxi Medical University, Guangxi Medical University, Nanning, China

**Keywords:** COVID-19, vaccine, efficacy, adverse event, meta-analysis

## Abstract

**Background:**

Inactivated vaccine is one of the primary technology types of Coronavirus Disease 2019 (COVID-19) vaccines, which has wide application in many countries, including mainland China. However, systematic evaluation of the efficacy and safety of COVID-19 inactivated vaccines remains limited. And trust in the vaccine is the key to solving vaccine hesitancy.

**Methods:**

Various academic databases were searched comprehensively for randomized controlled trials (RCTs) related to COVID-19 inactivated vaccines. The deadline for retrieval was December 2021. Study screening and data extraction were according to inclusive and exclusive criteria. Statistical analyses were performed using RevMan software 5.3 version and STATA software 16.0 version.

**Results:**

Eight studies with 79,334 subjects were included of which 48,123 had received two doses of COVID-19 inactivated vaccines, and 31,211 had received two doses of placebo. The results of the meta-analysis showed that: in terms of effectiveness evaluation, two doses of COVID-19 inactivated vaccines decreased the symptomatic infection [relative risk (RR) = 0.23, 95% confidence interval (CI) (0.18,0.30), *P* < 0.00001], asymptomatic infection [RR = 0.48, 95%CI (0.32, 0.74), *P* = 0.0008], total infection [RR = 0.32, 95%CI (0.24, 0.41), *P* < 0.00001] and hospitalization [RR = 0.06, 95%CI (0.01, 0.27), *P* = 0.0002] for severe acute respiratory syndrome coronavirus 2 (SARS-CoV-2) significantly. In terms of safety assessment, two doses of COVID-19 inactivated vaccines also caused more adverse events. After two inoculations, total adverse events and systemic adverse events increased significantly [total adverse events RR = 1.14, 95%CI (1.08, 1.21), *P* < 0.00001; systemic adverse events RR = 1.22, 95%CI (1.09, 1.35), *P* = 0.0002]. The most common adverse event was pain at the injection site. Almost all local adverse reactions consisted of these events. The incidence of pain at the injection site was related to adjuvants. Using aluminum hydroxide as an adjuvant increased local pain significantly [RR = 1.97, 95%CI (1.52, 2.55), *P* < 0.00001]. Two doses COVID-19 inactivated vaccines did not increase serious adverse events [RR = 0.71, 95%CI (0.57, 0.90), *P* = 0.004].

**Conclusion:**

Two doses of inactivated COVID-19 vaccines in people over 18 years of age effectively prevented SARS-CoV-2 infection and its associated hospitalizations. Short-term, mild to moderate adverse reactions had occurred, but serious adverse events were rare. No placebo or vaccine-related deaths had been reported.

**Systematic review registration:**

https://www.crd.york.ac.uk/prospero/, identifier: 42021291250.

## Introduction

Coronavirus disease 2019 (COVID-19), also known as novel corona pneumonia, is primarily transmitted via respiratory droplets and direct contact with susceptible humans and is highly contagious (1). Since the World Health Organization (WHO) declared COVID-19 as a global pandemic in 2020, the number of confirmed cases has been reported to be hundreds of millions, including millions of deaths (2). Consequently, for asymptomatic patients and non-specific symptomatic individuals with active SARS-CoV-2 infection, it is challenging to control and prevent the spread of the infection (3). In addition to the SARS-CoV-2 test, hygiene and physical distancing measures have been used to manage the disease, which has shaken economic activity, social interactions, work organizations, and how people live their daily lives in most countries. In this context, public attention to the COVID-19 vaccine has increased.

The foundation of primary healthcare is the safety and efficacy of various vaccines. Previously, raising awareness on vaccinations safeguarded many people from infectious diseases such as measles, tuberculosis, and poliomyelitis successfully (4). Meanwhile, the lack of concern and perception of the risk of infectious diseases has caused complacency, even making it a particular problem in the form of “vaccine hesitancy” (5). Vaccine hesitancy refers to the delay or refusing vaccines despite available vaccination services (6). Before the worldwide COVID-19 pandemic, vaccine hesitancy had become one of the top 10 threats to global health (7). Hesitancy to receive the vaccine is also a significant barrier to the COVID-19 vaccination. A meta-analysis of the intention to get vaccinated against COVID-19 in different countries revealed that COVID-19 vaccine hesitancy existed in 20% of adults [95% confidence interval (CI) (13, 29%)] (8). Vaccine hesitancy is a pivotal point in achieving high coverage in COVID-19 vaccination. An understanding of the 3Cs model on vaccine hesitancy, including confidence, complacency, and convenience, is critical in developing and implementing interventions for the problem. COVID-19 vaccine hesitancy is common the world over, and according to the model, building trust in COVID-19 vaccines is key to addressing this.

Recently, numerous studies have investigated the safety and efficacy of COVID-19 vaccines, most of which were about advanced technology platforms, like recombinant subunit proteins, virus-like particles, messenger RNA, DNA, and viral vectors (9, 10). Some countries, including mainland China, have used inactivated vaccines for emergency vaccination against COVID-19. Regardless, a systematic evaluation of the vaccines remains limited. We conducted a meta-analysis on the effectiveness and safety of the COVID-19 inactivated vaccine to provide helpful information about the COVID-19 vaccination.

## Materials and methods

### Search strategy

We registered the study under the prospective international register of systematic reviews [PROSPERO, registration ID: CRD42021291250]. We used the medical subject headings (MeSH) combined with random words to identify related research systematically in both Chinese and English databases. We also located additional studies by manually searching for relevant references. The date range for the meta-analysis included studies published from when the databases were established to December 2021.

### Study selection

The inclusion criteria were (a) The population uninfected by SARS-CoV-2 without age, ethnicity, and gender limitations. (b) Subjects were in a healthy state or a stable condition with chronic non-curable diseases such as cardiovascular disease and diabetes. (c) In the studies, subjects were divided into at least two groups, the intervention group, and the control group. (d) The interventions in intervention groups included receiving two doses of COVID-19 inactivated vaccines. (e) Moreover, control groups received at least two doses of placeboes. (f) The type of research was RCT. (g) At least one of the outcome indicators contained the following: ① effectiveness outcome measures: the numbers of individuals with SARS-CoV-2 infection confirmed by reverse transcription-polymerase chain reaction (RT-PCR) of nasal-pharyngeal swab after 2 weeks of receiving two-doses vaccines or placeboes, including asymptomatic individuals, symptomatic individuals, hospital admission or death. Safety outcome measures: the frequencies of adverse events after 4 weeks of receiving 2-doses of vaccines or placeboes, including total adverse events, pain around the injection site, systemic adverse reactions, and serious adverse events.

Exclusion criteria included: (a) Animal experiments. (b) Small sample size clinical trials. (c) Subjects had undergone any type of COVID-19 vaccine before participating. (d) Those with known allergies to any vaccine component or placebo. (e) Women who were breastfeeding, pregnant, or planning to become pregnant during the study. (f) Those with severe systemic diseases (uncontrolled hypertension, diabetes, cardiovascular disease, liver and kidney disease, malignancy, and autoimmune disease) that might interfere with the trial were excluded. (g) Those using immunosuppressive agents, corticosteroids, chemotherapy, and other therapies may alter their immune status. (h) If issues overlap, one of them would be ruled out. (i) If full-text articles or valid data were not accessible.

Initially, we used NoteExpress software to delete duplicate literature and skimmed the titles and abstracts to eliminate the literature which did not meet the inclusion criteria. Then, we read the full text for detailed information to determine its final inclusion. At this step, we recorded the reasons for rejecting particular studies.

### Data extraction

The data were extracted and reviewed by XL and XY. Extracted data included the first author, study location, study date, duration of follow-up, registration number, types of research, development corporation, intervention, comparison, the number of subjects and their baseline characteristics, outcome index, and so on. We set the possible sources of heterogeneity in the subgroup anteriorly: age, the health state and gender ratio of subjects, sample sizes, type of immunomodulators, and inoculation interval between two doses.

### Outcomes

The primary outcomes for effectiveness were the confirmed cases of SARS-CoV-2 infections. The confirmed cases were limited to at least 14 days after two vaccinations and confirmed by Rt-PCR in the laboratory, including symptomatic infections, asymptomatic infections, total infections, hospitalizations, or deaths.

Safety outcomes included local adverse reactions, systemic adverse reactions, and severe adverse reactions. Subjects were recorded and reported using the adverse reaction monitoring system 28 days after vaccination. Clinical trial observers rated association and severity based on the records. The same adverse reaction was recorded only once for the subject. When multiple symptoms were combined, each sign was registered once.

### Quality assessment

We used the Cochrane Handbook for Systematic Reviews Interventions to assess the risk of bias.

### Statistical analysis

All the variables of outcome in our study were dichotomous. Relative risk (RR) and 95% confidence interval (CI) were calculated as effect analysis statistics.

First, we conducted the heterogeneity test by *Q* statistic and *I*^2^ tests. The studies were homogeneous if the *Q*-test >0.10 and the *I*^2^ test < 50%. We applied the fixed-effects model to pool homogeneous studies. Otherwise, there was significant heterogeneity. Exploring the sources of heterogeneity was the next step. The random-effect model is generally used in such situations. Forest plots were applied for the analysis results. Finally, funnel plots were used for testing publication bias, while Begg's and Egger's methods were adopted for testing quantitative bias. It was considered publication bias when *P* ≤ 0.1.

We conducted the meta-analyses by using RevMan software [version 5.3]. Sensitivity analyses were performed in STATA software [version 16.0] to explore the cause of heterogeneity.

## Results

### Literature screening

**Figure 1:**
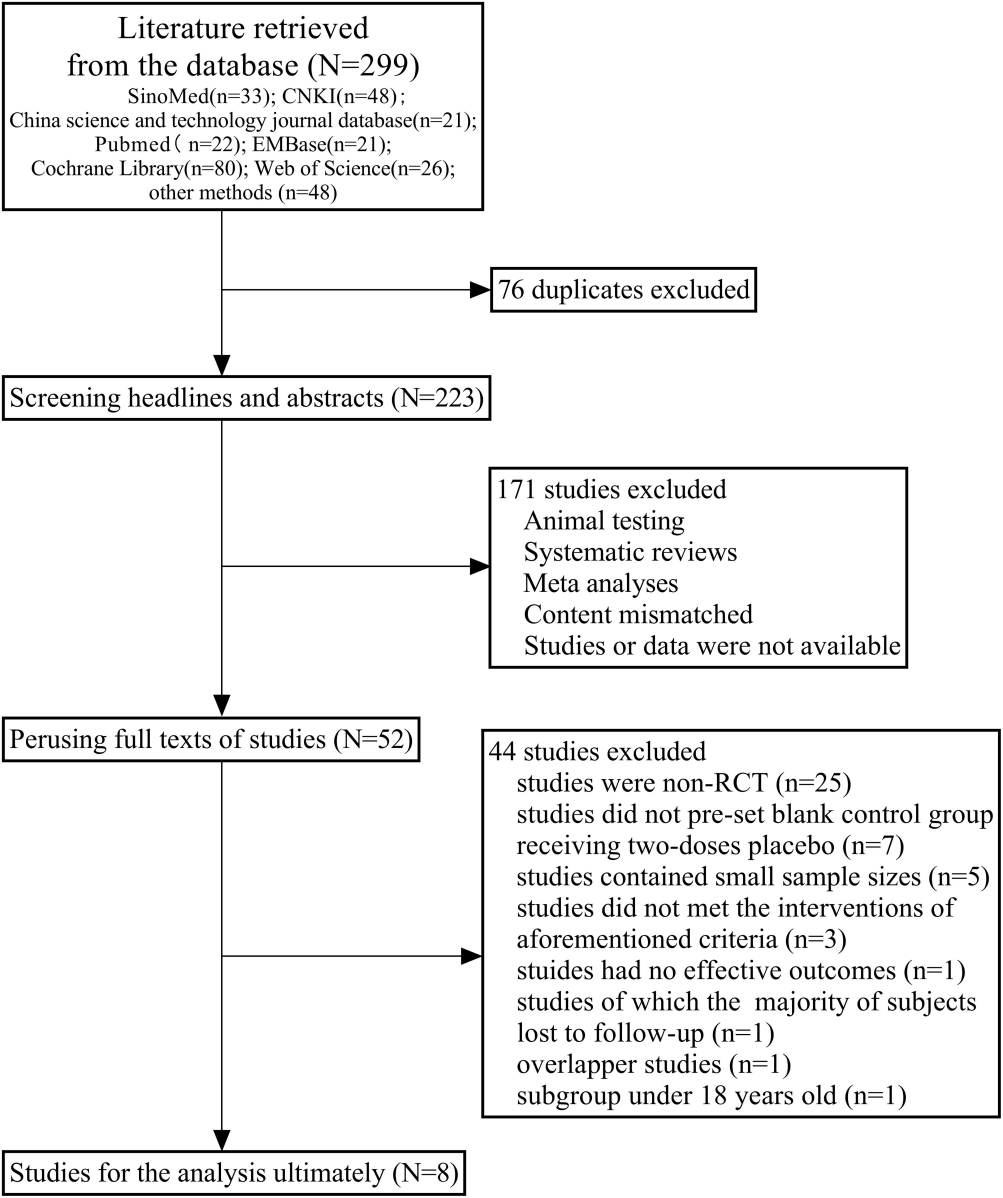
Flow chart of literature screening.

We obtained 299 studies [Sino Med, 33 studies; Chinese national knowledge infrastructure (CNKI), 48 studies; China science and technology journal database, 21 studies; PubMed, 22 studies; Embase, 21 studies; Cochrane library, 80 studies; web of science, 26 studies; and other sources/methods, 48 studies] initially by searching databases comprehensively or identifying from relevant references manually. In the first phase, we eliminated 76 duplicates and retained 223 studies. After that, we excluded 121 studies based on the abovementioned criteria of screening headlines and abstracts. One hundred and two studies were included after preliminary screening, of which 50 were excluded because of the lack of valid data or availability of full texts. Subsequently, from the 52 full texts of studies that we perused, we excluded 43 studies [seven studies did not pre-set blank control group of a placebo, five studies contained tiny sample sizes, 25 studies were non-RCT, three studies did not meet the intervention criteria mentioned earlier, one study had no effective outcomes described above, in one study the majority of the subjects were lost to follow-up, one of the two overlapped studies was excluded, and one study presented results for children and adolescents under 18 years old whose baseline characteristics of subjects differed from other studies]. We finally selected eight RCTs for the analysis (11–18). All the studies were in English and were RCTs that compared the safety and efficacy of two doses of inactivated COVID-19 vaccines with a placebo. The flow chart of the literature screening is shown in Figure 1.

### Methodological quality assessment

All eight included studies were placebo-controlled RCTs. The Ethics Committee had approved the protocols. Four studies were double-blind, multicenter trials (12, 13, 17, 18). Three studies were double-blind, single-center trials (14–16). One study was a single-blind, single-center trial (11). Only one of the eight studies did not describe the method of randomization or allocation concealment (18). In Mine et al.'s (12) study, the subjects were unblinded according to the requirements of the Ethics Committee because of the ministry of health in Turkey's emergency vaccination on 13 January 2021. Despite only the data before the cut-off date of unblinding being included in the final analysis, the risk of bias possibly existed. The remaining seven studies reported the results of trials as planned. Very few subjects were lost to follow-up or withdrawal. Most subjects of a study carried out in the Middle East were male because of local customs (13). The proportions of male participants in the two vaccine intervention groups and the placebo control group were 84.0, 84.5, and 84.8%, respectively. There was less likelihood of other biases existing in the remaining studies. We assessed the methodological quality of the eight included studies by the Cochrane Collaboration tool for risk of bias, as shown in Supplementary Figures S1, S2.

### Studies characteristics and baseline of subjects

The included eight RCTs involved five different inactivated vaccines, including CoronaVac developed by Sinovac Research and Development Co., Ltd (11, 12, 15, 16), BBIBP-CorV developed by Sinopharm (13), Inactivated SARS-CoV-2 vaccine (Vero cell) developed by Shenzhen Kangtai Biological Products Co., Ltd (14), BBV152 vaccine developed by Bharat Biotech International Limited (17), and SARS-CoV-2 vaccine (Vero cells) developed by Institute of Medical Biology and Chinese Academy of Medical Sciences (18). All the subjects received two doses of placebo or vaccines. Furthermore, the COVID-19 inactivated vaccines were stored at 2–8°C and transported by a cold chain. The method of inoculation was intramuscular injection at the deltoid. A single dose of the vaccine was 0.5 ml. The interval between the two doses of vaccination was 14 days, 21 days, or 28 days. Follow-up periods ranged from 56 days to 219 days. Four studies compared the effects of different dosages (13, 15, 16, 18).

Nevertheless, our meta-analysis only included the injection dosages in phase III clinical trials. Three studies compared the effects of various interval times (14, 15, 18). One study compared the differences between two inactivated vaccines (13). One study compared the different batches of the same vaccine (11). The information and characteristics of included RCTs are shown in Tables 1, 2.

**Table 1:** Characteristics of included studies.

**References**	**Vaccine name**	**Devlopers**	**Registration number**	**Phase**	**Research method**	**Adjuvants**
Eddy et al. (11)	CoronaVac	Sinovac Research and Development Co., Ltd	NCT04508075	III	Single center randomized single-blind control	Aluminum hydroxide
Mine et al. (12)	CoronaVac	Sinovac Research and Development Co., Ltd	NCT04582344	III	Multicenter randomized double-blind control	Aluminum hydroxide
Nawal et al. (13)	BBIBP-CorV	Sinopharm	NCT04510207 ChiCTR2000034780	III	Multicenter randomized double-blind control	Aluminum hydroxide
Hongxing et al. (14)	Inactivated SARS-CoV-2 vaccine (Vero cell)	Shenzhen Kangtai Biological Products Co., Ltd	ChiCTR2000039462	I/II	Single center randomized double-blind control	Aluminum hydroxide
Yanjun et al. (15)	CoronaVac	Sinovac Research and Development Co., Ltd	NCT04352608	I/II	Single center randomized double-blind control	Aluminum hydroxide
Zhiwei et al. (16)	CoronaVac	Sinovac Research and Development Co., Ltd	NCT04383574	I/II	Single center randomized double-blind control	Aluminum hydroxide
Raches et al. (17)	BBV152 vaccine	Bharat Biotech International Limited	NCT04641481	III	Multicenter randomized double-blind control	Algel-IMDG*
Yanchun et al. (18)	SARS-CoV-2 vaccine (Vero cells)	Institute of Medical Biology and Chinese Academy of Medical Sciences	NCT04412538	II	Multicenter randomized double-blind control	Aluminum hydroxide

^*^Algel-IMDG was Alum like receptor 7/8 agonist molecules adsorbed to the alum.

**Table 2:** Characteristics of included studies.

**References**	**Sample size**	**Dosage**	**Interval**	**Control measures**	**Follow-up time Effectiveness/safety**	**Outcomes**
Eddy et al. (11)	1,602	3 μg	0/14 days	Sterilized water for injection	104 days/194 days	
Mine et al. (12)	10,214	3 μg	0/14 days	Aluminum hydroxide 0.225 mg	48 days/121 days	
Nawal et al. (13)	40,388	5 μg 4 μg	0/21 days	Aluminum hydroxide 0.5 mg	156 days/167 days	
Hongxing et al. (14)	300	5 μg	0/14 days 0/28 days	Aluminum hydroxide 0.25 mg	—/56 days	
Yanjun et al. (15)	360	3 μg 6 μg	0/14 days 0/28 days	Aluminum hydroxide	—/56 days	
Zhiwei et al. (16)	199	1.5 μg 3 μg 6 μg	0/28 days	Aluminum hydroxide	—/219 days	
Raches et al. (17)	25,753	6 μg	0/28 days	Aluminum aseptic phosphate buffer	99 days/146 days	
Yanchun et al. (18)	450	100 EU 150 EU	0/14 days 0/28 days	Aluminum hydroxide	—/56 days	

Effectiveness outcome measures: symptomatic infection confirmed by RT-PCT; asymptomatic infection confirmed by RT-PCT; hospitalized due to SARS-CoV-2 infection; total adverse events at 28 days after two doses; pain events by injection at 28 days after two doses; systemic adverse events at 28 days after two doses; serious adverse events at 28 days after two doses.

We enrolled 79,334 subjects, of which 48,123 subjects were in the two doses COVID-19 vaccine intervention group, and 31,211 subjects were in the two doses placebo control group. All the subjects were from Asia. Their ages ranged from 18 to 97 years old. Two studies reported the results of COVID-19 inactivated vaccines in older adults over 60 years old (16, 17). Two studies enrolled the cohorts diagnosed with chronic non-communicable diseases (NCD) while being in normal immune function (12, 17). The baseline of the enrolled population is shown in Table 3.

**Table 3:** Baseline of the population.

**References**	**Area**	**Sample size intervention group/control group**	**Male/female**	**Age mean/range**	**BMI**	**Health state**
Eddy et al. (11)	Indonesia	798/804	1,046/574	35.5/18–59	24.65	Healthy
Mine et al. (12)	Turkey	6,646/3,568	5,907/4,307	45.0*^/^18–59	25.70*	Healthy or chronic NCD while being in normal immune function^#^
Nawal et al. (13)	Saudi Arabia Bahrain	26,935/13,453	37,594/612	36.1/≥18	27.00	Healthy
Hongxing et al. (14)	Jiangsu, China	200/100	135/191	44.0/18–59	–	Healthy
Yanjun et al. (15)	Jiangsu, China	240/120	169/191	41.9/18–59	–	Healthy
Zhiwei et al. (16)	Hebei, China	125/74	100/98	65.9/≥60	26.41	Healthy
Raches et al. (17)	India	12,879/12,874	17,285/8,468	40.1/18–97	24.30	Healthy or chronic NCD while being in normal immune function^#^
Yanchun et al. (18)	Yunnan, China	300/150	178/270	39.2/18–59	23.48	Healthy

^*^Median.

^#^Normal immune function referred to neither corticosteroids nor immunosuppressants.

### Outcomes of effectiveness

#### Preventing symptomatic infections

**Figure 2:**
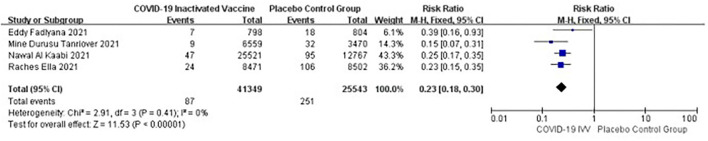
Forest plot of symptomatic infections.

Four studies [*n* = 66,892] (11–13, 17) included in the meta-analysis showed that two doses of COVID-19 inactivated vaccines decreased the symptomatic infections caused by SARS-CoV-2 [RR = 0.23, 95%CI (0.18, 0.30), *P* < 0.00001; Figure 2].

#### Preventing asymptomatic infections

**Figure 3:**
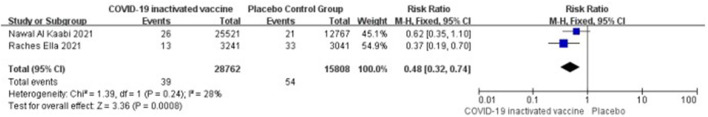
Forest plot of asymptomatic infections.

Two studies [*n* = 44,570] (13, 17) included in the meta-analysis showed that two doses of COVID-19 inactivated vaccines decreased the asymptomatic infections caused by SARS-CoV-2 [RR = 0.48, 95%CI (0.32, 0.74), *P* = 0.0008; Figure 3].

#### Preventing total infections

**Figure 4:**
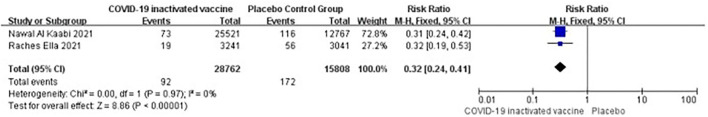
Forest plot of total infections.

Two studies [*n* = 44,570] (13, 17) included in the meta-analysis showed that two doses of COVID-19 inactivated vaccines decreased the SARS-CoV-2 infections [RR = 0.32, 95%CI (0.24, 0.41), *P* < 0.00001; Figure 4].

#### Preventing in-hospital events caused by SARS-CoV-2 infections

**Figure 5:**
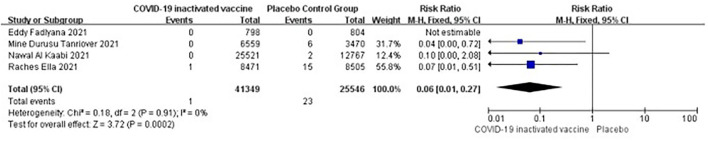
Forest plot of hospitalizations for SARS-CoV-2 infections.

Four studies [*n* = 66,895] (11–13, 17) included in the meta-analysis showed that two doses of COVID-19 inactivated vaccines decreased the in-hospital events caused by SARS-CoV-2 infections [RR = 0.06, 95%CI (0.01, 0.27), *P* = 0.0002; Figure 5].

### Outcomes of safety

#### Total adverse events

**Figure 6:**
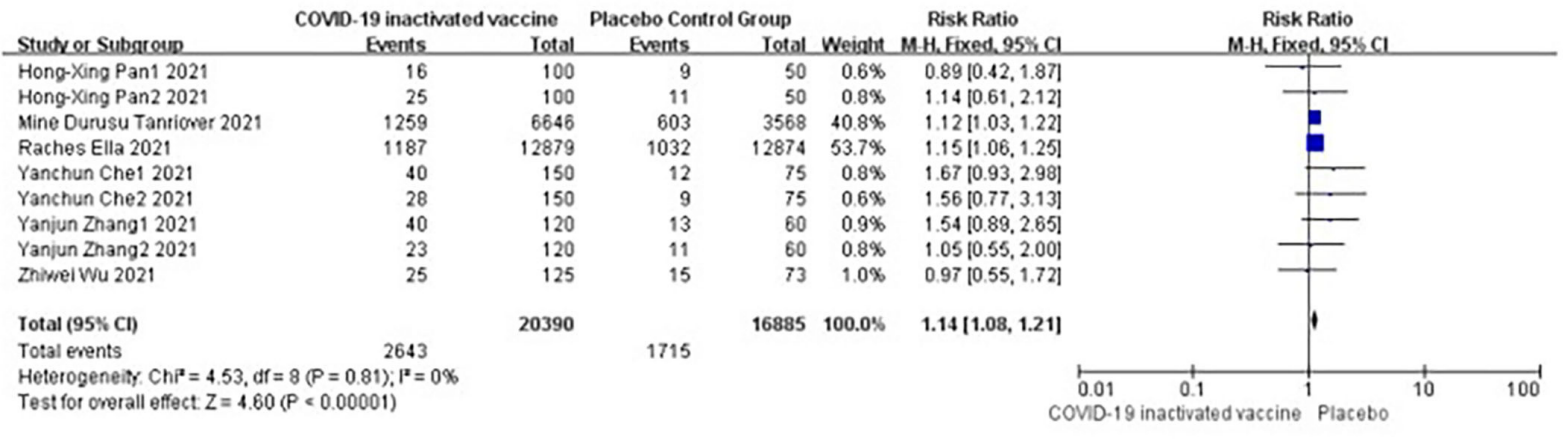
Forest plot of total adverse events.

**Figure 7:**
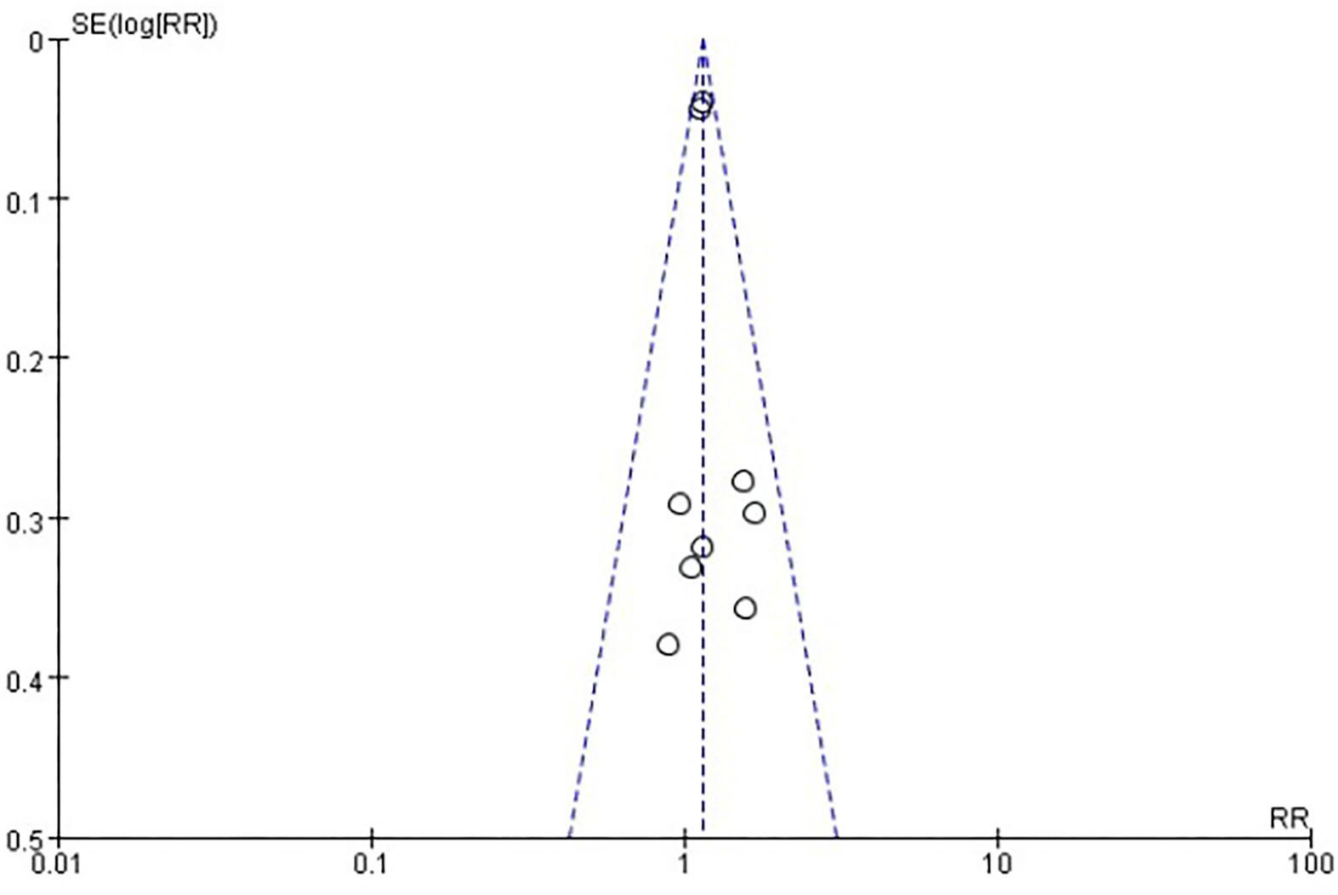
Funnel plot of total adverse events.

Six studies [*n* = 37,275] (12, 14–18) included in the meta-analysis showed that two doses of COVID-19 inactivated vaccines increased total adverse events [RR = 1.14, 95%CI (1.08, 1.21), *P* < 0.00001; Figure 6]. No publication bias was found visually or statistically by funnel plots (Figure 7), Egger's test [Pr > |*z*| = 0.677], or Begg's test [*P* > |*t*| = 0.434].

#### Systemic adverse events

**Figure 8:**
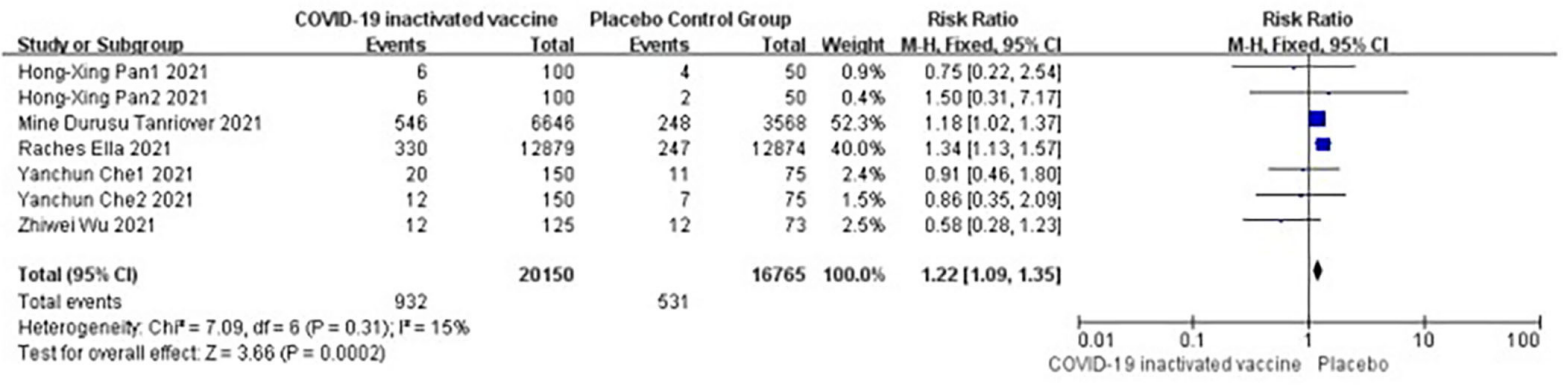
Forest plot of systemic adverse events.

Five studies [*n* = 36,915] (12, 14, 16–18) included in the meta-analysis showed that doses of COVID-19 inactivated vaccines increased systemic adverse events [RR = 1.22, 95%CI (1.09, 1.35), *P* = 0.0002; Figure 8].

#### Adverse events of pain at injection site

**Figure 9:**
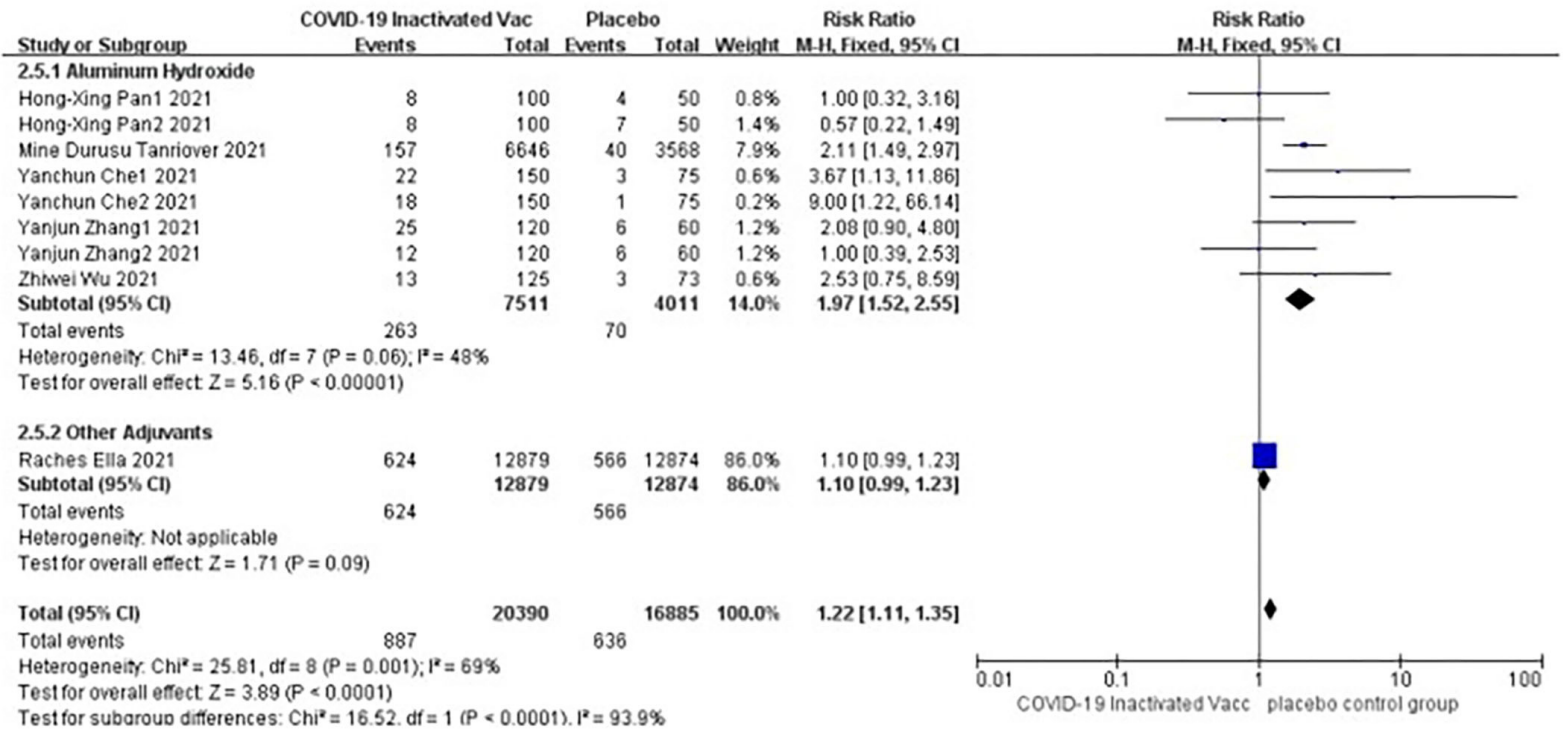
Forest plot of pain at injection site.

**Figure 10:**
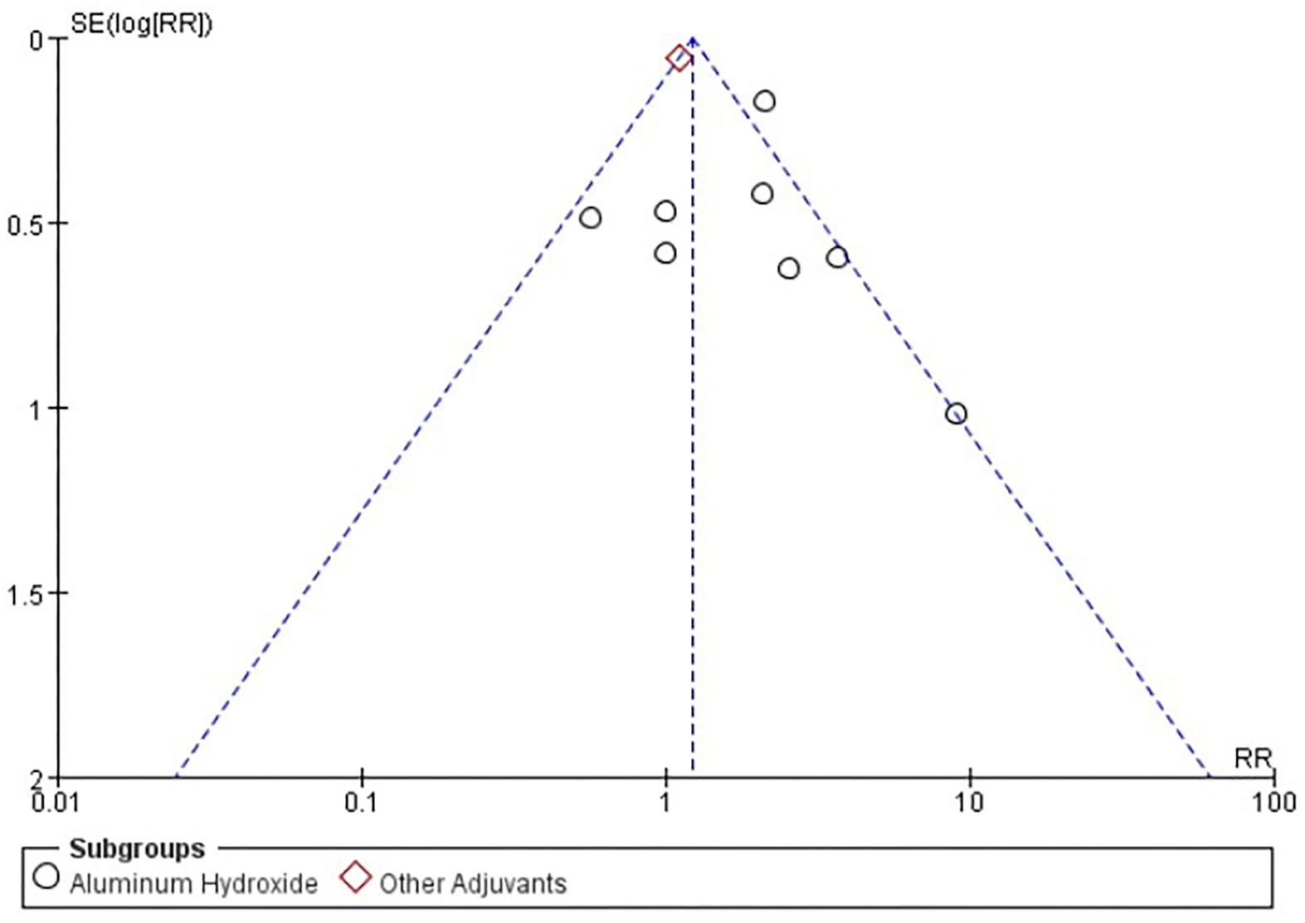
Funnel plot of pain at injection site.

Six studies [*n* = 37,275] (12, 14–18) included in the meta-analysis showed a significant difference between the subgroup of adjuvants. In the aluminum hydroxide subgroup, pain at injection site events increased significantly [RR = 1.97, 95%CI (1.52, 2.55), *P* < 0.00001; Figure 9]. No publication bias was found visually or statistically by funnel plots (Figure 10), Egger's test [Pr > |*z*| = 0.297], or Begg's test [*P* > |*t*| = 0.158].

#### Serious adverse events

**Figure 11:**
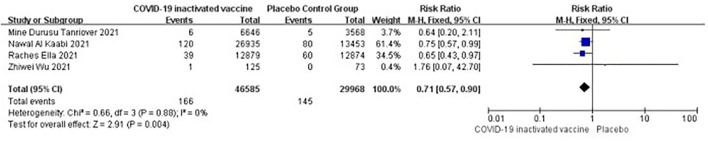
Forest plot of serious adverse events.

Four studies [*n* = 36,915] (12, 13, 16, 17) included in the meta-analysis showed that two doses of COVID-19 inactivated vaccines did not trigger serious adverse events [RR = 0.71, 95%CI (0.57, 0.90), *P* = 0.004; Figure 11).

A total of four vaccine-related serious adverse events were reported in the included studies. A female subject suffered a grade 3 systemic allergic reaction after inoculation, as reported in Mine et al.'s study (12). A seronegative issue for SARS-CoV-2 at baseline occurred in immune thrombocytic purpura after vaccination, as reported in Ella et al.'s study (17). In Nawal et al.'s study, there were two serious adverse events related to the vaccine in the HB02 vaccine group. A 30-year-old male subject who was heterozygous for a very long-chain acyl-CoA dehydrogenase deficiency variant suffered acute demyelinating encephalomyelitis (ADEM) after the first inoculation. A 35-year-old female subject suffered severe vomiting after the second inoculation (13). All the subjects who suffered extreme adverse events improved after treatment.

#### Cases of mortality

Only Ella et al.'s study reported 15 mortality cases considered unrelated to the vaccine or placebo. Five belonged to the vaccine group (cerebellar hemorrhage, hemorrhagic stroke, ovarian cancer metastasis, cardiac arrest, and COVID-19), and the others were in the placebo-control group (alcohol excess, myocardial infarction, cardiac arrest with hypertension, five subjects died from COVID-19 and two issues died by unknown cause) (17).

## Discussions

Some studies have approved new technology-platform COVID-19 vaccines (9, 19). There are still controversies about COVID-19 inactivated vaccines. The meta-analysis by Ali et al. showed that the short-term conversion rate of SARS-CoV-2 stereospecific antibodies after the COVID-19 inactivated vaccine was about 94%. The conversion rate of specific serum-neutralizing antibodies was < 80% (9). The results made it controversial. Whether a suboptimal humoral immune response implies a reduction in vaccine efficacy remains unknown. The acceptance of COVID-19 inactivated vaccines is rare in many countries. However, the inactivated vaccine may be the only available COVID-19 vaccine to the population of less developed areas. Moreover, mistrust of COVID-19 vaccines hinders vaccination.

Our study showed that two doses of COVID-19 inactivated vaccines decreased SARS-CoV-2 infections and their severity. In our study, all subjects with normal immune function were enrolled. Factors such as age, comorbidities, or gender had little effect on the effectiveness outcome. Other studies on COVID-19 vaccination in people with malignancies or the need for renal replacement therapy (RRT) pointed out that the immune status of the recipients may be the main factor affecting the effectiveness of vaccination (20–23). These special populations were immunosuppressed due to the use of immunotherapy or chemotherapy. It also attenuated vaccine-induced immune responses. The results of Ma et al.'s (20) study showed that the serum conversion rate of the special populations was significantly lower than that of the healthy people. Seyed et al. (21) obtained similar results in the population with malignant tumors. There is also a question of whether the immunological results fully represent the vaccine's protective effect. Maryam et al. (22) reported that although breast cancer patients receiving trastuzumab or chemotherapy had a significantly reduced immune response to the inactivated COVID-19 vaccine BBIBP-CorV, the vaccine still provided adequate protection during short-term follow-up. Such a thing might also exist in immunocompetent people. The immune response induced by vaccination is complex. Most studies on inactivated COVID-19 vaccines assessed only parts of the humoral immune response. To some extent, this represented the effect of the vaccine, but denying the protective impact of inactivated vaccines might be one-sided. More extensive studies were needed for a full assessment.

The occurrence of adverse events increased after inoculation when the vaccines were effective. Most of the adverse events were mild to moderate and relieved in the short term. Only a small number of subjects reported grade three or higher adverse events (the incidence of the vaccine group was 0.3%, and the placebo group was 0.4%). The results of our safety review suggest that the vaccine is tolerable in an immunocompetent population. Safety reviews of COVID-19 vaccines by Nadim Sharif et al. showed similar results (19). Pain at injection site events was the most common adverse event. In the subgroup analysis, aluminum hydroxide used as an adjuvant appeared to cause more pain events. There was a difference between the aluminum hydroxide subgroup and the Algel-IMDG subgroup. However, the number of studies included in the Algel-IMDG subgroup was limited. More studies were still needed to demonstrate this issue. Some inactivated vaccines use aluminum hydroxide as an adjuvant to strengthen the immunization to achieve the expected effect currently. And a few reports indicated that aluminum hydroxide was associated with comorbidities after inoculation. Previously, pancreatitis had been considered to induce by aluminum hydroxide possibly used in other inactivated vaccines (24, 25). Recent reports speculated that serious adverse events such as Bell's palsy, type 1 Kounis syndrome, chronic inflammatory demyelinating polyneuropathy, and hepatitis after receiving COVID-19 inactivated vaccine might be the results of interaction with genetic susceptibility and aluminum hydroxide (26–29). There have also been reports of psoriasis, herpes zoster, or induction of secondary retinal changes after the COVID-19 inactivated vaccine (30–34). Many questions remain unanswered. It is still unclear whether the events correlated with aluminum hydroxide or whether a new type of adjuvant was superior to aluminum hydroxide. Nevertheless, these case reports should arouse enough attention from clinicians. In addition, Maryam et al. (22) and Mona et al. (23) reported similar safety results for BBIP-CORV from Sinopharm Vaccinating in special populations such as patients with malignant tumors. And not just for COVID-19 inactivated vaccines, the review by Seyed et al. (21) also affirmed the safety of different types of COVID-19 vaccines for patients with malignancies. But weakened immune responses after vaccination make these special populations more vulnerable to an outbreak. Perhaps in the premise of guaranteeing security, exploring personalized immunization programs is a potential path for different groups of people. There is still a long way to go.

Most of the adverse events were mild or moderate, and subjects recovered in a short time. Only a tiny proportion of subjects reported severe adverse events. Two doses of COVID-19 inactivated vaccine could improve the severity and clinical outcome related to SARS-CoV-2 infection at the same time. Most populations benefited from the COVID-19 vaccination after trading potential adverse effects and benefits. Even though the COVID-19 vaccine did not end the global pandemic, it still played an essential role in alleviating the shock to healthcare systems. It would be unwise to reject COVID-19 inactivated vaccine while new technology-platform COVID-19 vaccines were unavailable.

## Limitations

The present research contains some limitations. Firstly, most of the subjects enrolled in our meta-analysis were male. The results may therefore be subject to sex bias. Secondly, because of the limited data, we could not perform a subgroup analysis in different circulating SARS-CoV-2 variant strains. Vaccine efficacy against different variant strains had a significant differentiation (17). The result is not unimportant to develop better vaccination strategies for adapting continuously changing epidemic strains. Finally, homologous, or heterologous boosts have been tested in some areas, which showed more robust protection compared with basal inoculation of two doses of COVID-19 inactivated vaccine, especially against epidemic SARS-CoV-2 variant strains. Regrettably, we did not conduct a subgroup meta-analysis due to a limited number of publications.

## Conclusion

Two doses of inactivated COVID-19 vaccines in people over 18 years effectively prevented SARS-CoV-2 infection and its associated hospitalizations. Short-term, mild to moderate adverse reactions had occurred, but serious adverse events were rare. No placebo or vaccine-related deaths had been reported.

## Data availability statement

The original contributions presented in the study are included in the article/Supplementary material, further inquiries can be directed to the corresponding author.

## Author contributions

XL, XY, and ZN contributed to conception and design of the study. XL performed the statistical analysis and wrote the first draft of the manuscript. XY and ZN wrote sections of the manuscript. All authors contributed to manuscript revision, read, and approved the submitted version.

## Conflict of interest

The authors declare that the research was conducted in the absence of any commercial or financial relationships that could be construed as a potential conflict of interest.

## Publisher's note

All claims expressed in this article are solely those of the authors and do not necessarily represent those of their affiliated organizations, or those of the publisher, the editors and the reviewers. Any product that may be evaluated in this article, or claim that may be made by its manufacturer, is not guaranteed or endorsed by the publisher.
