# Supplementary figures and images for: Efficacy and safety of COVID-19 inactivated vaccine: A meta-analysis
Source: Front Med (Lausanne). 2022 Nov 7;9:1015184. doi: 10.3389/fmed.2022.1015184 (PMC9676443; doi:10.3389/fmed.2022.1015184)

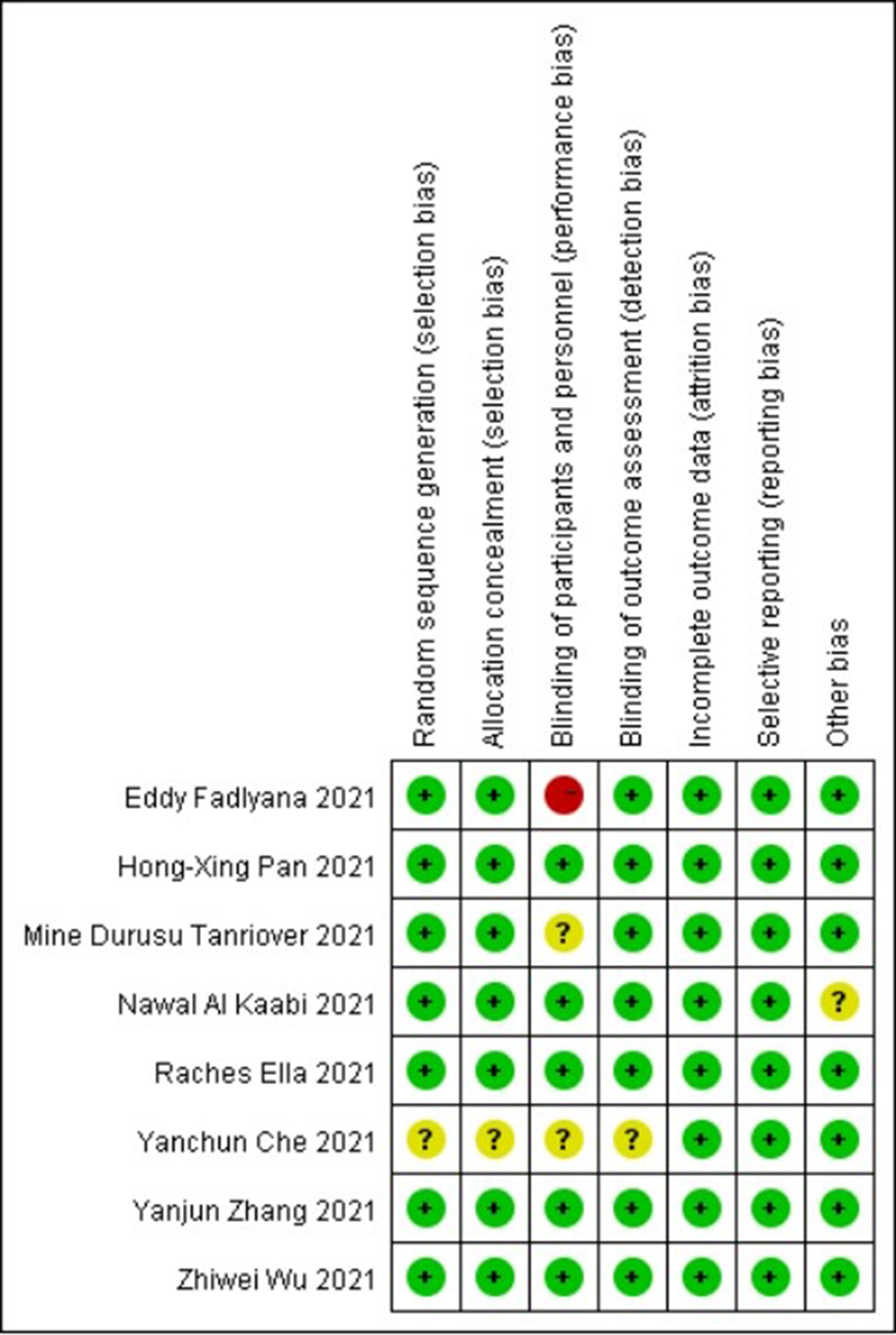

Supplement: Supplementary file 1 [file Image_1.JPEG]

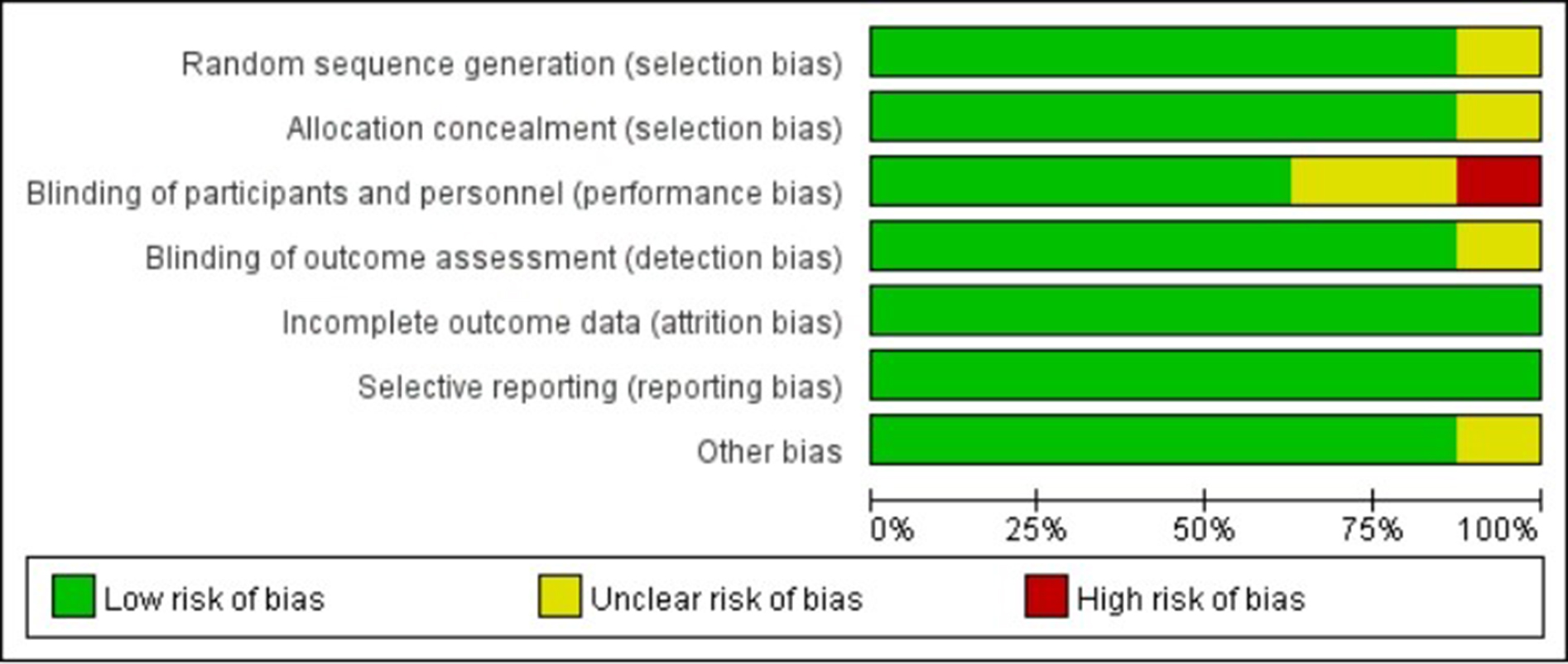

Supplement: Supplementary file 2 [file Image_2.JPEG]
